# Effect of Honey Bee Colony Strength on Foraging Productivity and Its Application to Precision Pollination

**DOI:** 10.3390/insects17020163

**Published:** 2026-02-02

**Authors:** Sandra Kordić Evans, George Clouston, Yuval Regev, Elizabeth M. Walsh, Kate Ihle, Frank Rinkevich, Michael Simone-Finstrom, Huw Evans

**Affiliations:** 1 BeeHero Inc., Palo Alto, CA 94036, USA; huw@beehero.io; 2 BeeHero Ltd., Tel Aviv 6511703, Israelyuval@beehero.io (Y.R.); 3 USDA-ARS Honey Bee Breeding, Genetics, and Physiology Unit, 1157 Ben Hur Rd, Baton Rouge, LA 70806, USAkate.ihle@usda.gov (K.I.);

**Keywords:** honey bees, colony strength, foraging productivity, pollination efficiency, precision pollination

## Abstract

Honey bee pollination of entomophilous commercial crops is a major input in agricultural management yet unlike irrigation, fertilisation and plant protection have yet to be integrated into precision agriculture practices. This study examines colony strength as a key determinant of efficient pollination. Over three years and across two study sites, we evaluated the relationship between colony strength (frames of bees, FOBs) and colony productivity using continuous hive weight monitoring. Hive weight data were analysed for both absolute gains and relative gains normalised per FOB across colony strengths. In all study periods, stronger colonies showed disproportionately higher weight gains compared to weaker colonies. For each additional FOB, the average increase in normalised weight gain ranged from 0.1 to 0.41 kg per colony, indicating a non-linear relationship between colony strength and productivity. An efficiency factor calculated for groups of strong and weak colonies ranged from 1.2 to 2.6, depending on the season and crop. Moreover, during periods of forage dearth, strong colonies exhibited lower weight losses than the weak colonies per FOB, making them more efficient under resource limited conditions. Our findings demonstrate that colony strength significantly influences foraging efficiency and colony resilience, ultimately supporting the conclusion that fewer stronger colonies will improve pollination outcomes while reducing the economic and environmental costs associated with commercial pollination services.

## Simple Summary

Commercial pollination services are a multi-million dollar industry in the United States and are growing in other regions of the world as the intensive cultivation of high value crops such as almonds and blueberries increases. This growth has prompted research into precision pollination approaches, analogous to those used in precision agriculture, with the aim of optimising the use of honey bee colonies and other resources. Honey bee colonies are often transported across large geographical distances to crops and this practice impacts both the economics of pollination service and the environment. In this study, we examined whether pollination services can be streamlined by using strong colonies rather than larger numbers of weaker colonies. We found that strong colonies consistently and disproportionately outperform weak colonies during pollination and are also more resilient in periods of dearth. Prioritising the deployment of stronger colonies is proposed in order to improve pollination outcomes and beekeepers’ revenues and to reduce the number of colonies that are transported, thus also lowering the environmental impact of the transhumance.

## 1. Introduction

Honey bee pollination plays a central role in modern agricultural production, particularly for high-value pollinator-dependent crops. Since the 1960s, agricultural land area dedicated to pollinator-dependent crops has expanded globally and more rapidly than that of non-dependent crops [1,2,3,4]. Agricultural intensification has led to an increase in field size and the widespread simplification of agricultural habitats, resulting in extensive cultivated areas with low abundance of wild pollinators [5,6,7,8]. In the United States, Europe, and increasingly in Australia, pollinator-dependent crops rely predominantly on managed pollinators, with the western honey bee (*Apis mellifera*) accounting for the vast majority of pollination services [9,10,11,12,13]. Parallel to this growth in managed pollinator dependence, financial investment in pollination services has risen sharply, reflecting both the value and vulnerability of these systems. Globally, pollinator-dependent crops are valued at USD 235 to USD 577 billion annually, and they also play a crucial role in sustaining global food security [11].

Among pollinator-dependent crops, almonds, apples, blueberries, and avocados have expanded most rapidly in recent decades [14,15]. Almond production in California provides the clearest illustration of the scale, complexity, and economic significance of managed pollination. Over 80% of the world’s almonds are produced in California, where more than 2.7 million honey bee colonies are transported every winter to pollinate over 1.4 million acres of orchards [16,17,18]. Similar expansion is occurring in Australia, where more than 300,000 colonies are mobilised each season for almond pollination [19]. These large-scale pollination events come at a substantial economic and environmental cost as the provision of pollinating units and transport costs rise, while honey bee colonies frequently suffer significant stress and mortality due to long-distance transportation [20].

These challenges raise the question of whether optimising managed pollination, through the use of fewer, stronger colonies, could reduce pressures on honey bee populations and, in turn, benefit beekeepers, growers, and ecosystems. Even modest improvements in pollination efficiency, could have substantial net effects, given the scale of operations. These improvements would translate into increased economic sustainability, reduced carbon emissions, and potentially improved honey bee colony health. Addressing this question requires a better understanding of the factors that drive colony pollination efficiencies and performance under real-life pollination and production cycles. While irrigation, nutrition, plant protection, and other agricultural inputs have advanced substantially under the umbrella of precision agriculture, managed pollination has not kept pace. As a biological process delivered by managed pollinators, it remains among the least optimised components of modern agriculture [21], despite the fact that in some production systems, such as almonds, pollination services account for a significant proportion of operational costs [22]. Optimising pollination is inherently challenging because managed pollinators are not an ecologically inert agricultural input; they rely on the very ecosystem resources they help sustain [23]. While this presents another challenge, it also provides an opportunity to improve colony state and, consequently, the efficiency of managed pollinators. Numerous initiatives have emerged within agro-ecosystems to support managed pollinators, such as planting cover crops in almonds, which carry financial incentives for landowners by offering alternative forage for bees [17]. For a comprehensive review of approaches to safeguarding pollinators (both wild and managed) in agro-ecosystems, see [13].

In this agricultural framework, the goal of optimising managed pollination is to address the parameters governing optimal colony performance. These include colony strength, stocking density, hive placement, and timing of deployment, each of which can contribute to effective pollination with optimal use of resources. Precision pollination therefore represents a key shift toward a more sustainable, data-driven management of an essential ecosystem service. However, implementing such an approach requires a robust quantitative understanding of how colony performance scales with strength, environmental conditions, genetics, and management practices.

In a previous study examining the effect of hive orientation on the activity of honey bees during almond pollination [24], we found that colony strength alone was sufficient to shift the activity dynamics of colonies facing an unfavourable western exposure. This prompted further interest in quantifying the other benefits of using strong colonies. Anecdotal beekeeping evidence supports the idea that stronger colonies are disproportionately more efficient for honey production. Scientific evidence, however, is not fully conclusive despite almost a century of research. Numerous studies [25,26,27,28,29,30,31,32] have examined how colony size, demography, and queen quality influence honey production and colony efficiency. While substantial evidence supports stronger colonies outperforming weaker ones, not all studies substantiate this relationship. Determining whether, and under what conditions, colony performance exhibits increasing, constant, or diminishing returns with colony size will help elucidate the scaling of this relationship and its relevance for modern pollination systems.

In this study, we revisit these long-standing questions using continuous field data obtained by monitoring hive weights and assessing colony strength across multiple crops, seasons, and landscapes. We evaluate whether increases in colony performance scale linearly with strength or exhibit characteristics of an economy of scale, where stronger colonies yield disproportionately higher returns. We examine how the state of the colony—defined by its strength, structure, queen age, and condition—relates to foraging and pollination activity under realistic field conditions. By quantifying these relationships, this research aims to contribute to the development of more efficient, resilient, and sustainable managed pollination systems for precision pollination in modern agriculture.

## 2. Materials and Methods

### 2.1. Study Sites and Honey Bee Stock

Honey bee colonies were studied in two distinct geographical regions, both characterised by a Mediterranean climate. Because colony foraging performance is strongly influenced by weather, floral phenology, and landscape context, data were collected across four field studies spanning multiple crops, landscapes, and years. Specifically, two studies were conducted during almond (*Prunus dulcis*) pollination in Central California in 2023 and 2024, one study during *Robinia pseudoacacia* flowering in Central Italy in spring 2025, and one study during *Castanea sativa* flowering in Central Italy in summer 2025. Each study represents a distinct combination of site, crop, season, and year, allowing us to test whether the relationship between colony strength and foraging productivity is consistent across contrasting ecological and management conditions rather than confined to a single site or season.

Study site 1 was located in the Central Valley of California in a commercial almond (*Prunus dulcis* Miller) orchard (almond cultivars: 50% Nonpareil, 25% Aldrich and 25% Monterey) in Tranquillity, CA, USA (Fresno County, CA, USA, 36.65029° N, 120.27688° W). The site lies at an elevation of 50 m a.s.l. The area is characterised by an intensive agricultural landscape, predominantly dedicated to large-scale monocultures of almonds and pistachios.

In 2023, 24 honey bee colonies representing three different genetic lines of *Apis mellifera* (Russian, Pol-line, and unselected) were monitored during almond pollination from 17 February to 15 March. Both Pol-line and Russian bees were selectively bred by USDA–ARS for Varroa mite resistance and raised by the same beekeeper, while unselected stock were raised by a different beekeeper. Each colony was housed in two-deep 10-frame Langstroth boxes.

In 2024, 16 honey bee colonies of unselected stock were brought to the site on 13 February and were subject to comparable phenological conditions as in 2023. Eight colonies were housed in one-deep and one-medium 10-frame Langstroth boxes stacked; this group was removed from the site on 27 February, so their monitoring period runs only from 15 to 27 February. The remaining eight colonies were housed in two-deep 10-frame Langstroth boxes and were monitored from 15 February to 15 March. In both years, half of the colonies had their entrances facing west and the other half east; however, hive entrance orientation was not included as a fixed effect in the present analysis, as it was examined separately in a previous almond pollination study.

Study site 2 was situated in an established apiary in Tuscany, Central Italy (Bagni di Lucca, LU, Italy, 44.03572° N, 10.58273° E). The site lies at an elevation of 340 m a.s.l. in the foothills of the Apennine mountains. The area is dominated by dense sweet chestnut (*Castanea sativa*) forests and mixed woodlands.

In 2025, 10 honey bee colonies from a local stock of *Apis mellifera ligustica* were monitored. First study was from 23 April to 9 May during *Robinia pseudoacacia* (hereafter Robinia) flowering, and the second from 1 June to 30 June during the flowering of *Castanea sativa* (hereafter sweet chestnut). All colonies were housed in 10-frame Dadant–Blatt boxes, and honey supers (half the height of the brood box) were added and removed following standard beekeeping practices during nectar flow. All colonies had their entrances facing south; hence, orientation was not included as a fixed effect in the present analysis.

### 2.2. Electronic Equipment

In all trials, the bee hives were placed on proprietary hive scales (developed by BeeHero Inc., Palo Alto, CA, USA). Each scale used four 40 kg load cells, one at each corner, sandwiched between two 6 mm aluminium plates to mitigate uneven loads. The resulting maximum capacity of the scale was 160 kg, with a precision of ±30 g and an operating temperature range of −20 to +60 °C. The output from the load cells was fed to a 12-bit ADC input of the microcontroller via a low-noise amplifier. This signal was upsampled to 16-bit over a period of 250 ms. The scale was powered by two ICR14500 3.7 V 1.6 mAh onboard lithium-ion batteries connected in parallel. The internal batteries were supplemented using a 24 Wh/6700 mAh lithium-ion “always-on” power bank combined with a 5 W solar panel.

A single weather pack was installed at a central location near the bee hives in all four trials. It measured ambient temperature (±0.5 °C accuracy) and rainfall using a self-emptying tipping-bucket rain gauge, allowing classification of rainfall intensity. Unlike fixed-chamber rain gauges, a self-emptying gauge does not require manual emptying. The unit was powered using an LP952040 800 mAh lithium-polymer battery, providing several months of operation, and was charged via an onboard USB port.

Synchronised samples from both the hive scales and the weather pack were taken every 5 min and periodically uploaded to the communication gateway via Bluetooth. The communication gateway stored this data locally and uploaded it to the server/cloud every 30 min via the 4G LTE cellular network.

In the 2024 almond study, pollinator activity in the orchard was monitored from 17 February to 8 March, using small acoustic sensors (developed by BeeHero Inc., USA) trained to detect honey bee flight noise. In-field sensors were powered by specialised batteries with a limited operational lifespan and were therefore deployed during the most critical period of interest, namely the almond bloom, to maximise data collection during peak pollination activity. The accuracy of this system has been shown to correlate well with manual counts (R^2^ = 0.81) [33]. A total of 200 sensors were installed across two almond cultivars; 25 sensors per row across 8 rows, resulting in a monitored area approximately 50 m wide and extending 50 m into the orchard from the bee hives. The sensors formed a proprietary mesh topology that relayed data back to the communication gateway using Bluetooth communication.

### 2.3. Honey Bee Colony Strength

In all four studies, honey bee colonies were assessed for strength using primary metrics for population (adult bees and brood) and secondary metrics for stores (honey and pollen). All strength assessments involved opening the hives and removing individual frames for visual assessment. In the 2023 almond and 2025 Robinia and sweet chestnut studies, assessments were performed twice, at the beginning and end of the study, using the established Liebefeld method [34] which provides a detailed and repeatable assessment of colony strength and offers a good balance between speed and accuracy. In the 2024 almond study, colony strength was also assessed twice, at the beginning and end of the study, but this time using a standard beekeeper inspection. This is a faster and more approximate method that reflects routine practice in large-scale commercial pollination settings where rapid assessment of many colonies is required. Both approaches were applied consistently within each study to categorise colonies.

For each colony, weight gain was calculated as the difference between end and start hive weights when there was an overall increase in hive weight. Analogously, weight loss was calculated as the difference between end and start hive weights when there was an overall decrease. Weight values used to calculate gains and losses were taken from the local midnight readings. Hive weight data are used as a proxy to represent overall colony productivity, including stored honey as well as adult bees, brood, pollen, wax, and hive hardware.

Across all studies, colonies exhibited varying strengths, representing realistic field conditions and enabling categorisation into Weak and Strong groups for analysis. Division was based on the median colony strength (frames of bees, FOBs). Colonies with FOB values less than or equal to the median were classified as Weak, while those with FOB values greater than the median were classified as Strong.

### 2.4. Statistical Analysis

Relationships between colony strength (FOB) and weight gain or loss were evaluated using simple linear regression. Weight change was analysed both as absolute gain or loss and as normalised gain or loss per unit strength (kg FOB^−1^). In all regressions, colony strength (FOB) measured at the beginning of the experiment was treated as a continuous predictor, and weight change as the response variable. Effect sizes were quantified using the regression slope (kg FOB^−1^) and the coefficient of determination (R^2^). Statistical significance was assessed using *p*-values associated with the regression coefficient. These analyses were conducted on ungrouped colony data to quantify the strength and direction of the relationship between colony strength and weight change within each study.

Differences between Strong and Weak groups were tested using non-parametric Mann–Whitney U tests (two-tailed, α = 0.05), as group sizes were small and weight gain data per group were not normally distributed. Statistical comparisons were performed on absolute weight gains, not efficiency ratios, to avoid ratio variance artefacts arising from variability in the denominator (FOB), which can artificially increase variance and affect the interpretation of group differences.

All statistical calculations were conducted in Microsoft Excel (Microsoft 365 for Mac, version 16.94). The raw datasets used in these analyses are provided in the Supplementary Materials (Supplementary File S1).

## 3. Results

Results are presented from two different sites and across three years. The study sites differed in both landscape and resource availability and the study years varied in ambient conditions. The environmental contexts form the basis for the interpretation of the site-specific and inter-annual observations described below.

During the study periods, colony weight changes primarily reflected foraging activity on the target plants. In the Italian studies, the target species (*Robinia pseudoacacia* and *Castanea sativa*) flowered during distinct, temporally separated periods during which honey bees predominantly exploited these abundant floral resources. The floral origin of harvested honey was identified based on characteristic organoleptic properties (e.g., colour, aroma, and crystallisation behaviour) and beekeeper experience, indicating the dominance of the target crops. In the California studies, colonies were placed in a predominantly monoculture agricultural landscape. While alternative floral resources were present in 2023, these did not overlap temporally with the almond flowering period. Subsequently, colony weight gains observed during almond bloom can be attributed primarily to foraging on the target crop (*Prunus dulcis*), driven mainly by colony expansion and brood production.

### 3.1. Almonds 2023 (Central California)

**Figure 1:**
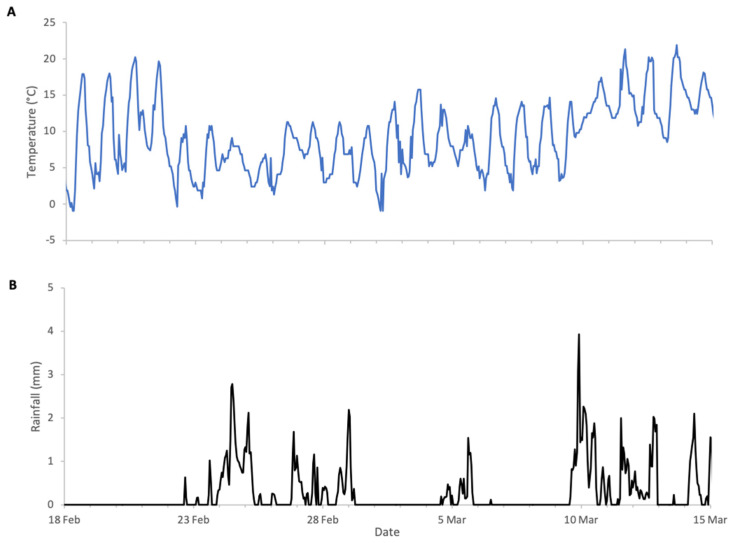
Weather conditions during almonds 2023 study: (**A**) ambient temperature, (**B**) rainfall.

**Figure 2:**
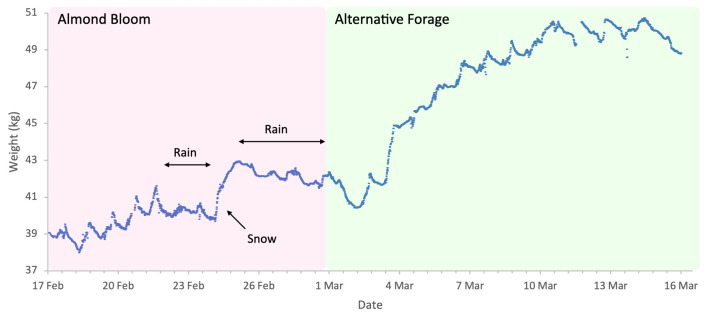
Sample hive weight data for a colony during the almonds 2023 study.

Almond bloom in 2023 was characterised by unfavourable foraging conditions (Figure 1) with low ambient temperatures and frequent, abundant precipitation particularly during peak bloom (18 to 27 February field visual observations) including snowfall on 24 of February. This event is clearly visible in Figure 2, as it produced an atypical increase in hive weight, even in the absence of bee activity. Although more favourable weather followed, most almond bloom had already finished. Nonetheless, we observed significant post-bloom colony weight increases after 4 March 2023 (Figure 2), which coincided with the availability of alternative forage resulting from agricultural management decisions on surrounding farms where neighbouring landowners established flowering cover crops.

#### Weight Gains

Over the entire study period, 17 February to 15 March 2023, stronger colonies gained more weight in absolute terms irrespective of their genetic line. However, linear regression of normalised weight gain per unit strength (FOB) across all colonies did not show a strong relationship between colony strength and weight gain (R^2^ = 0.07, *p* = 0.21).

**Figure 3:**
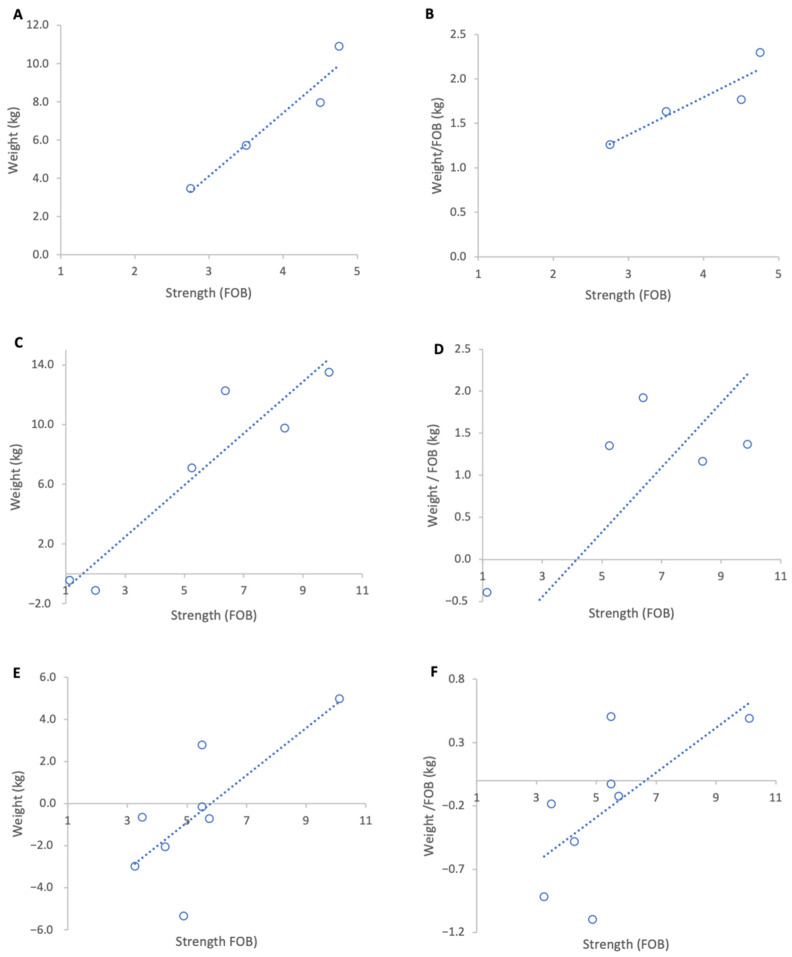
Relationship between colony strength and colony weight gain for three genetic lines from 17 February to 15 March 2023. (**A**) Pol-line total weight gain, (**B**) Pol-line weight gain normalised per FOB, (**C**) Russian total weight gain, (**D**) Russian weight gain normalised per FOB, (**E**) stock total weight gain, (**F**) stock weight gain normalised per FOB. Circles represent individual colony data points; dotted lines indicate fitted linear relationships.

When genetic lines were analysed separately, Pol-line colonies exhibited the highest normalised weight gains (Figure 3B) and a strong positive, but non-significant association between colony strength and weight per FOB (Figure 3A), likely due to the low sample size (R^2^ = 0.82, *p* = 0.09). This group originally contained eight colonies but for analysis four colonies were removed as they had been relocated on the day of grading leading to a significant loss of foragers which disrupted the baseline ratio of FOB to weight. Russian colonies achieved the highest absolute weight gains (Figure 3C) and a moderate but statistically significant positive relationship between colony strength and normalised weight gain (R^2^ = 0.64, *p* = 0.01) (Figure 3D). Unselected stock colonies either lost weight or only showed marginal gains during the course of study resulting in weaker associations between colony strength and weight gain per FOB (R^2^ = 0.43, *p* = 0.08) (Figure 3F).

Across genetic lines, the average increase in normalised weight gain per additional FOB was 0.41 kg in Pol-line colonies, 0.36 kg in Russian colonies, and 0.17 kg in unselected stock.

### 3.2. Almonds 2024 (Central California)

**Figure 4:**
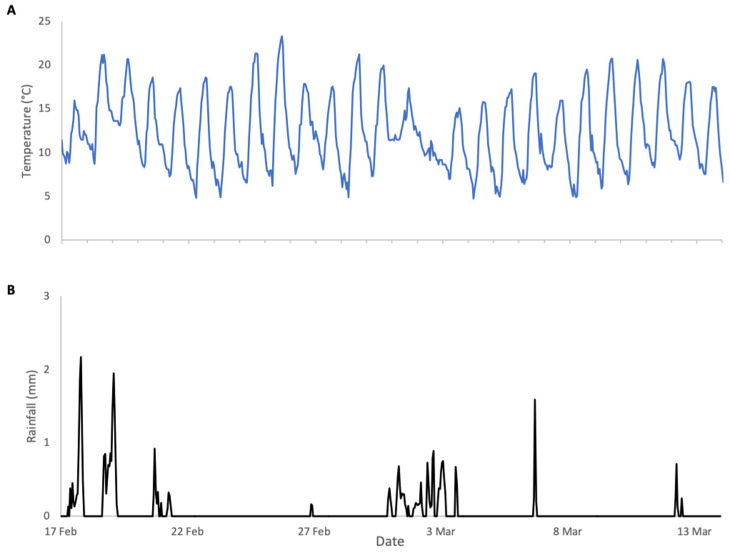
Weather conditions during almonds 2024 study: (**A**) ambient temperature, (**B**) rainfall.

**Figure 5:**
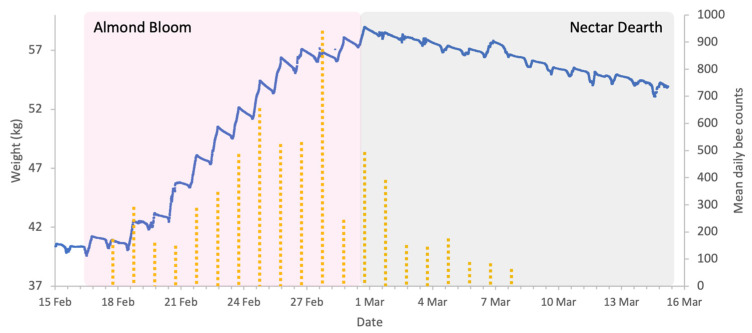
Sample hive weight data for a colony (blue line) and mean honey bee activity in almond orchard (yellow dotted columns) during the almonds 2024 study. Acoustic sensors register honey bee visitation events.

In contrast to 2023, the weather conditions during 2024 almond pollination were very favourable for both bloom phenology and bee activity (Figure 4). Daytime temperatures were consistently above 12 °C, the lower limit for bee activity, and nighttime lows were within a range that did not compromise almond pollen production. Rainfall was intermittent and considerably lower than in 2023 with minimal impact on bee activity. As a result, colonies exhibited substantial weight increases during bloom (Figure 5), followed by clear weight losses once bloom ended. In contrast to 2023, the surrounding fields in 2024 were planted with crops that did not provide floral resources during the study period, resulting in a pronounced post-bloom forage dearth. Accordingly, weight gains and losses were analysed as separate periods.

In this study, the deployment of in-field sensor (IFS) mesh showed that honey bee presence in the almond orchard was strongly influenced by the meteorological conditions and almond forage (Figure 5). Bee activity was consistently high from mid-February to 2 March 2024 except during rainfall. After 2 March, activity declines significantly, corresponding to the end of bloom. The weight increase on 7 March (Figure 5) is a result of rain rather than incoming forage.

#### 3.2.1. Weight Gains

**Figure 6:**
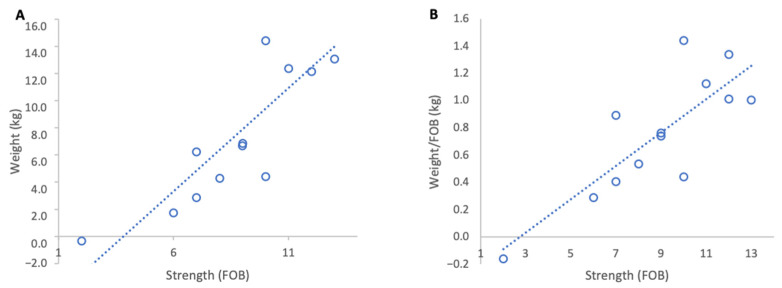
Relationship between colony strength and colony weight gain from 18 to 27 February 2024: (**A**) total weight gain per colony, (**B**) weight gain normalised per FOB. Circles represent individual colony data points; dotted lines indicate fitted linear relationships.

During the almond bloom period from 18 to 27 February 2024, stronger colonies gained more weight in absolute terms and colony strength showed a strong positive relationship with absolute weight gain (R^2^ = 0.74, *p* < 0.001) (Figure 6A). When normalised per FOB, the relationship remained significant and moderately strong (R^2^ = 0.66, *p* < 0.001). The regression slope indicated that each additional FOB contributed 0.12 kg of normalised weight gain (Figure 6B).

#### 3.2.2. Weight Losses

**Figure 7:**
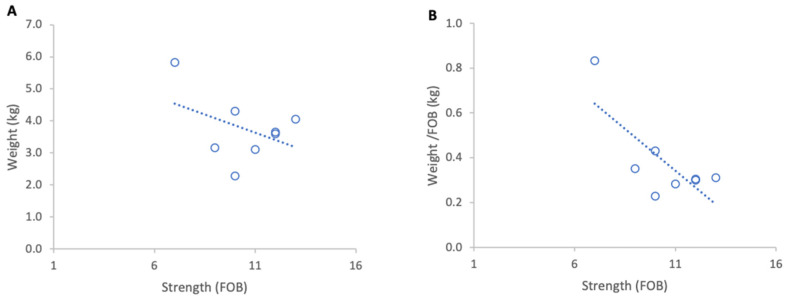
Relationship between colony strength and colony weight loss from 4 to 14 March 2024: (**A**) total weight loss per colony, (**B**) weight loss normalised per FOB. Circles represent individual colony data points; dotted lines indicate fitted linear relationships.

Following almond bloom, 4 to 14 March 2024, all colonies experienced weight loss. Absolute weight losses were similar across colonies of different strengths (R^2^ = 0.17, *p* = 0.31) indicating a weak relationship between colony strength and total weight loss (Figure 7A). However, when normalised per FOB there was a significant negative relationship (R^2^ = 0.56 *p* = 0.031 Figure 7B). Stronger colonies lost less weight relative to their strength than weak colonies. This suggests that stronger colonies are more resilient to dearth conditions.

### 3.3. Robinia 2025 (Central Italy)

**Figure 8:**
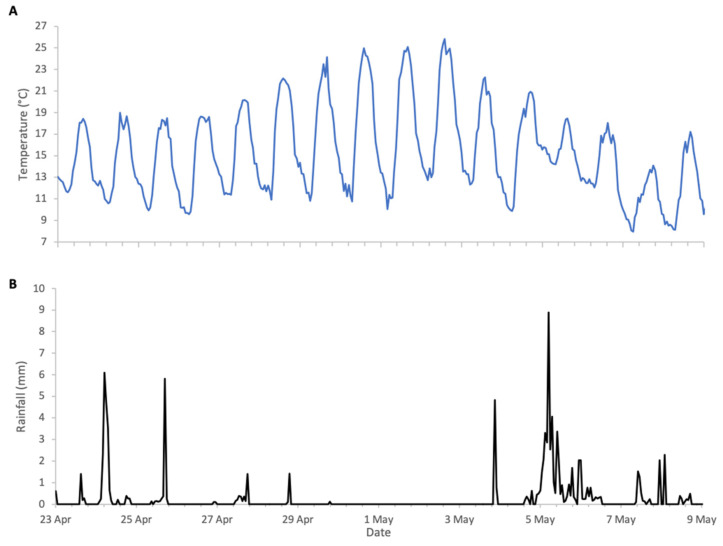
Weather conditions during Robinia 2025 study: (**A**) ambient temperature, (**B**) rainfall.

**Figure 9:**
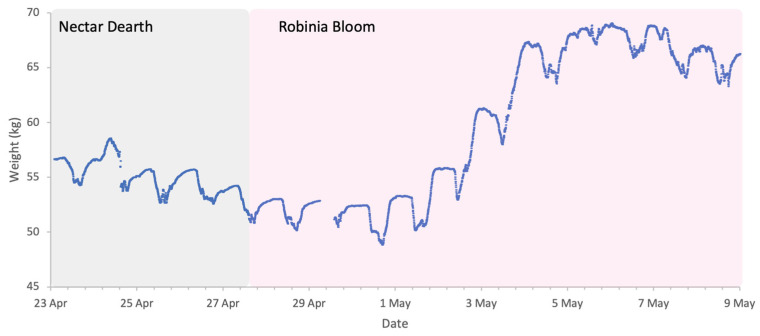
Sample hive weight data for a colony during the Robinia 2025 study.

Flowering of Robinia in 2025 was in line with its phenological trends of the last decade, spanning a period of approximately two weeks from late April to early May. The meteorological conditions, shown in Figure 8, were typical for the season, with temperatures generally exceeding 10 °C, the lower threshold for Robinia nectar secretion. The first half of bloom had intermittent precipitation, but colonies were active during dry spells and each accumulated in excess of 10 kg of weight during the two-week period (Figure 9).

#### 3.3.1. Weight Gains

**Figure 10:**
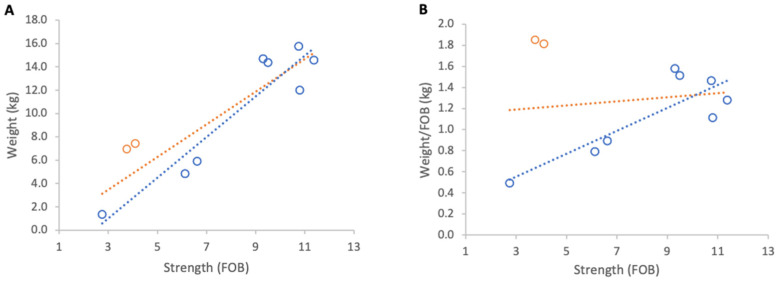
Relationship between colony strength and colony weight gain from 23 April to 9 May 2025: (**A**) total weight gain per colony, (**B**) weight gain normalised per FOB. Circles represent individual colony data points; blue circles indicate colonies headed by one-year-old queens and orange circles indicate colonies headed by same-season queens. Dotted lines indicate fitted linear relationships; the orange dotted line includes all colonies, whereas the blue dotted line excludes colonies headed by same-season queens.

Absolute weight gain per colony from 23 April to 9 May 2025 showed a strong positive relationship with colony strength (R^2^ = 0.79, *p* < 0.001; Figure 10A). However, normalised per FOB, the relationship was negligible and non-significant (R^2^ = 0.02, *p* 0,69; Figure 10B).

Further analysis showed that the two colonies created that spring and headed by new queens showed unusually high relative gains and were therefore distinct from the remainder of the group. When these colonies were excluded, relationships strengthened markedly for absolute and normalised gains (R^2^ = 0.88, *p* < 0.001; R^2^ = 0.70, *p* = 0.009, respectively). The average increase in normalised weight gain per additional FOB, obtained from the regression slope, corresponded to 0.1 kg of weight for each additional FOB (Figure 10).

#### 3.3.2. Weight Losses

**Figure 11:**
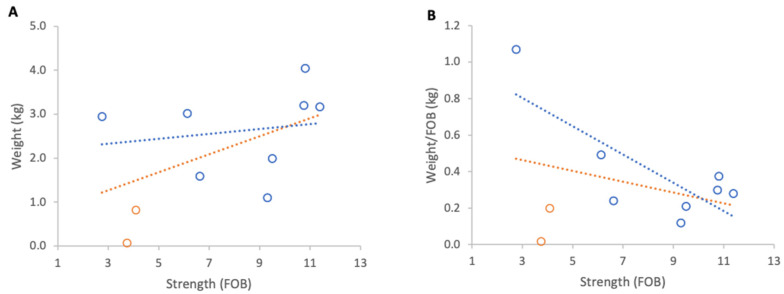
Relationship between colony strength and colony weight loss from 23 April to 29 April 2025: (**A**) total weight loss per colony, (**B**) weight loss normalised per FOB. Circles represent individual colony data points; blue circles indicate colonies headed by one-year-old queens and orange circles indicate colonies headed by same-season queens. Dotted lines indicate fitted linear relationships; the orange dotted line includes all colonies, whereas the blue dotted line excludes colonies headed by same-season queens.

Inclement weather at the beginning of nectar flow from 23 to 29 April 2025 resulted in a consistent overall loss of colony weights. When absolute weight loss was analysed, stronger colonies lost marginally more weight than weaker ones, although this relationship was not statistically significant (Figure 11A). When losses were normalised per FOB, the direction of the relationship reversed but remained statistically non-significant (Figure 11B).

Excluding the two colonies with same season queens, which showed that colony strength did not affect the absolute weight loss, there was a significant relationship between normalised weight loss and FOB (R^2^ = 0.60, *p* = 0.02).

### 3.4. Sweet Chestnut 2025 (Central Italy)

**Figure 12:**
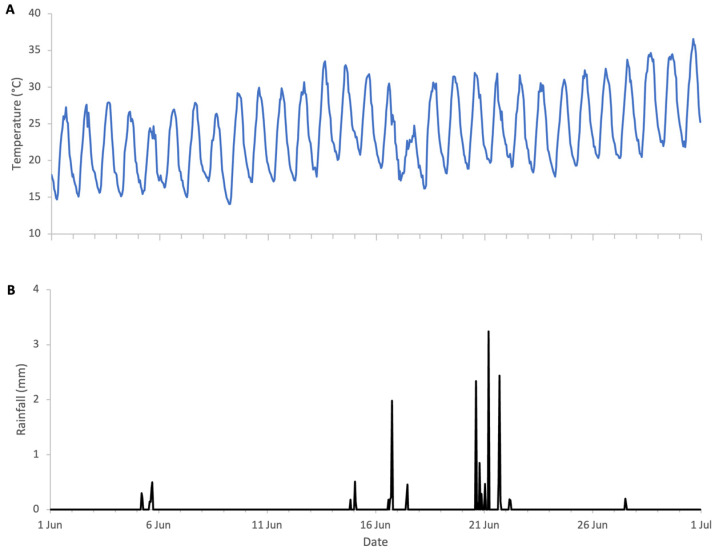
Weather conditions during sweet chestnut 2025 study: (**A**) ambient temperature, (**B**) rainfall.

**Figure 13:**
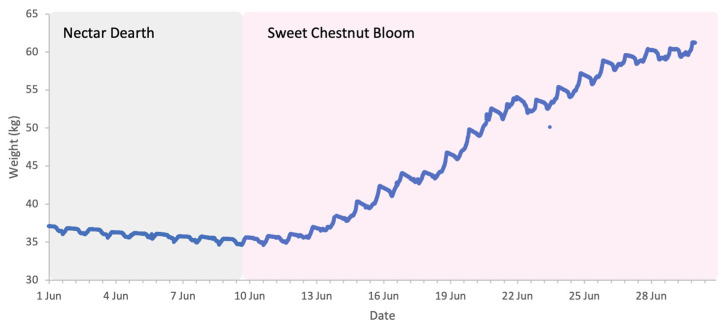
Sample hive weight data for a colony during the sweet chestnut 2025 study.

The flowering of sweet chestnut in Central Italy spans a period of approximately three to four weeks, due to overlapping phenology among locally adapted chestnut varieties and to some extent the altitudinal variation across the study area. The meteorological conditions during the period of sweet chestnut flowering were typical for early summer, with daytime highs of 30 to 35 °C, nighttime lows of 15–20 °C, and brief and light rainfall on 17 and 21 June. These conditions favour high honey bee foraging activity with minimal interruption (Figure 12). Colony weight data were analysed for the whole month of June and segregated into weight gains and weight losses as there was a distinct separation of two trends (Figure 13).

#### 3.4.1. Weight Gains

**Figure 14:**
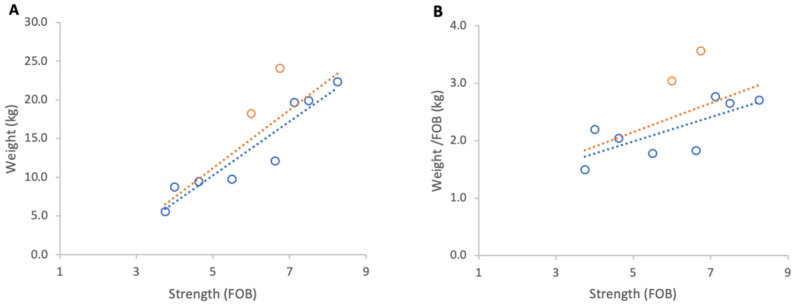
Relationship between colony strength and colony weight gain from 1 June to 30 June 2025: (**A**) total weight gain per colony, (**B**) weight gain normalised per FOB. Circles represent individual colony data points; blue circles indicate colonies headed by one-year-old queens and orange circles indicate colonies headed by same-season queens. Dotted lines indicate fitted linear relationships; the orange dotted line includes all colonies, whereas the blue dotted line excludes colonies headed by same-season queens.

All colonies accumulated substantial weight during chestnut bloom. Absolute weight gain from 1 to 30 June 2025 showed a strong and statistically significant positive relationship with colony strength (R^2^ = 0.76, *p* < 0.001; Figure 14A). When normalised per FOB, the trend indicated that stronger colonies gained more weight per FOB than weaker ones, although the relationship was not statistically significant (R^2^ = 0.35, *p* = 0.069; Figure 14B). However, when colonies headed by the current year queens were excluded from the analysis, both relationships strengthened considerably; absolute gains: R^2^ = 0.89, *p* < 0.001, compared to normalised gains: R^2^ = 0.62, *p* = 0.02. The regression slope indicated that each additional FOB contributed 0.25 kg of normalised weight gain, one of the strongest scaling effects observed across all trials.

#### 3.4.2. Weight Losses

**Figure 15:**
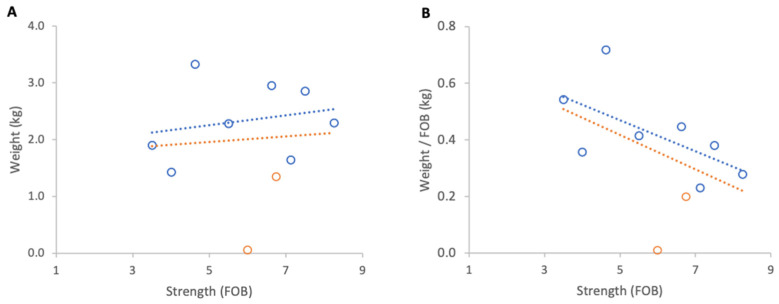
Relationship between colony strength and colony weight gain from 1 to 7 June 2025: (**A**) total weight loss per colony, (**B**) weight loss normalised per FOB. Circles represent individual colony data points; blue circles indicate colonies headed by one-year-old queens and orange circles indicate colonies headed by same-season queens. Dotted lines indicate fitted linear relationships; the orange dotted line includes all colonies, whereas the blue dotted line excludes colonies headed by same-season queens.

Prior to the start of sweet chestnut bloom, from 1 to 7 June 2025, there was a short dearth in forage and all colonies exhibited a net loss in weight. Absolute losses showed no relationship with colony strength (Figure 15A) indicating colonies of different sizes lost similar total weights.

However, when losses were normalised per FOB, a clear trend emerged: strong colonies lost less weight per FOB than weaker colonies (Figure 15B), though the result was not statistically significant. Excluding the colonies headed by new queens yields a stronger relationship but still not statistically significant (R^2^ = 0.38, *p* = 0.102).

### 3.5. Comparative Colony Efficiencies

To quantify the differences between Strong and Weak colony groups in each study, a comparative assessment of colony-level efficiency was performed. Table 1 summarises mean colony strength (FOB) and corresponding weight gains for each group and study period.

Group differences were statistically significant in the Robinia, sweet chestnut, and almond 2024 studies and marginally non-significant in the almond 2023 study. When normalised per unit colony strength (FOB), Strong colonies were consistently more efficient than Weak ones across all study periods, gaining 2.63, 2.19, 1.19/1.91, and 1.3 times more weight per FOB in almonds (2023), almonds (2024), Robinia, and sweet chestnut studies, respectively.

## 4. Discussion

Using colony hive weights and standard methods for assessing colony strength, our data showed that colony strength is a positive predictor of colony efficiency. Across three seasons, multiple forage sources, and contrasting landscapes, stronger colonies gained more weight per unit strength (FOB) than weaker colonies under the same environmental conditions. Furthermore, stronger colonies typically lost less weight per FOB than weaker colonies during forage dearths. In mass-flowering crops such as Robinia and sweet chestnut, concentrated nectar and pollen resources are preferentially exploited by honey bees, leading to strong foraging fidelity during bloom; under these conditions, colony weight gain serves as a robust proxy for colony-level foraging performance on the target crop.

### 4.1. Colony Strength and Foraging Performance

It is not unexpected that stronger colonies gain more overall weight than weaker colonies, as they inherently have larger forager populations. Studies have shown that the percentage of foragers in honey bee colonies remains constant across population sizes [25,35,36]. However, our analyses showed that Strong colonies consistently outperformed Weak colonies on a per-FOB basis, indicating a non-linear scaling of foraging productivity.

During the summer sweet chestnut bloom, a frame of bees in a Strong colony was 1.3 times more efficient than a frame of bees in a Weak colony. Similarly, during the preceding Robinia bloom, stronger colonies demonstrated 1.19 times greater efficiency, rising to 1.91 times when colonies headed by same season queens were excluded from the analysis. These findings broadly agree with previous studies: Farrar reported that colonies of 30,000, 45,000, and 60,000 bees produced 1.38-, 1.48-, and 1.54-fold more honey, respectively, than colonies of 15,000 bees [37]. Taranov’s studies from 1952, as reported by Woyke [29], found 1.42- and 1.70-fold increases in honey yield from colonies containing 2 kg and 4 kg of bees compared to 1 kg colonies.

Although Szabo and Lefkovitch [31] and Zacepins [32] do not explicitly report proportional honey yields, calculations derived from their published data indicate comparable efficiency ratios for stronger colonies: 1.3- and 1.41-fold, respectively.

In our late winter almond studies in 2023 and 2024, efficiency ratios were substantially higher, at 2.63 and 2.19, respectively. Given the distinct seasonal difference in the two study sites, our results suggest that the efficiency benefits seen in stronger colonies are greater earlier in the season, potentially due to ambient conditions imposing greater thermoregulatory demands on the colonies. In a previous study on hive orientation in almonds [24], we found that stronger colonies had a significant behavioural advantage over weaker colonies at threshold ambient temperatures; they commenced flight earlier in the day and maintained overall greater levels of activity than weaker colonies. This advantage was partly offset by hive orientation, with east-facing colonies becoming active earlier than those facing west. While the orientation in Robinia and sweet chestnut was identical for all hives (south-facing), in both almond studies the pallet configuration dictated an east/west orientation, likely amplifying the observed differences in net weight changes between Strong and Weak colonies. A number of other studies that investigated pollen collection rather than colony weight increase also support the idea of increasing returns with colony size [25,30,38,39]. In contrast, some investigators report absent or inconsistent evidence for this pattern [28,29].

The behavioural mechanisms that lead to stronger colonies being more efficient are well documented and include both individual forager and whole colony behaviour. Larger colonies exhibit a positive correlation with both the number of foragers and the effort each forager devotes to the task; individual foragers from larger colonies have been shown to fly further and carry heavier loads [40,41]. Donaldson-Matasci proposed that the competitive advantage conferred to large colonies arises from a greater net nectar gain per foraging trip than in smaller colonies, with activity peaking earlier in the day [42]. At first glance, this may appear to contrast with studies by Gary and Danka, which found that foraging activity increased linearly with colony size [43,44]. However, those studies do not report the loads carried by foragers from different-sized colonies, a factor that could tip the balance towards a non-linear relationship.

It is important to note that foraging activity cannot be assumed to be directly proportional to foraging productivity. Indeed, some of the earliest studies on this topic by Farrar [25,37] indicate that foraging activity increases proportionately with colony size, while weight gains show disproportionate increases with size. Conversely, Michener concluded that efficiency decreases with increasing colony size [45], although a recent meta-analysis of Michener’s original data indicates the opposite trend, which supports our findings [46].

Therefore, while it is not unequivocal that the efficiency benefits associated with stronger colonies apply universally, our results, along with substantial supporting research, indicate a consistent trend that has direct implications for improving the efficiency and outcomes of commercial pollination services as well as honey production.

### 4.2. Colony Performance at the Upper End of Colony Sizes

In three out of four studies, we observed convergence of weight gain values among the strongest colonies, suggesting that increases in strength at the upper end of the range do not translate into proportionally higher gains per FOB. In the 2024 almonds, 2025 Robinia, and 2025 sweet chestnut studies, both absolute and relative weight gains remained comparable in the top quartile of hive strengths. In the 2023 almond study, this effect is seen clearly in the Russian group of colonies, which comprised equal numbers of Weak and Strong colonies. A tail-off in efficiency was not observed in the Pol-line group, as the colonies had not yet reached their optimum strength. It is difficult to assess the existence of this trend in the stock group, as strength/efficiency correlations were much weaker than in the other groups.

Harbo also reported diminishing returns for the strongest colonies, where absolute weight gain was highest, but when adjusted to per-bee gain, showed little advantage compared to medium-sized colonies [26]. In contrast, the results presented by Zacepins, who evaluated colonies in a high-production area in Latvia, showed no slowing of relative weight gain with increasing colony size [32]. Given the disparate ranges of sizes, and perhaps more importantly the methods of estimating colony sizes, it remains unclear whether this trend is a consequence of colony size or other environmental factors, such as resource availability and meteorological conditions.

What could explain these diminishing returns? Congestion within stronger colonies can increase the time it takes for receiver bees to take and store loads from returning foragers [47]. In addition, queen pheromone, which has been shown to stimulate foraging [48], may circulate less efficiently in very large colonies. Large colonies may also have redundant stores and a higher brood-to-bees ratio than smaller colonies, reducing the stimulus to forage [31,39,49]. Finally, Free and Preece proposed that this effect may be caused by the cessation of colony growth, an explanation that could account for our summer-season results but does not explain the effect seen in almonds, where colonies had not yet reached their maximum populations [50].

Taken together, while diminishing returns were not universal, they appeared consistently across multiple contexts in our dataset and aligned with mechanistic expectations. The results suggest that while increasing colony strength confers clear efficiency benefits, these advantages are not indefinite and may stabilise once colonies reach high population thresholds. This has important implications for optimising—not simply maximising—colony strength in commercial pollination systems.

### 4.3. Effects of Genetic Line

The inclusion of three distinct genetic lines in the 2023 almond study allowed us to evaluate potential differences in productivity attributable to genetic background. Pol-line colonies showed a significant performance advantage over unselected stock and a small advantage over the Russian colonies.

Our findings align with Meikle et al., who reported that Pol-line and Russian lines outperformed unselected Italian stock bees across several performance criteria, including increased weight gain during nectar flow, decreased weight loss during dearth, and lower Varroa infestation levels [51]. This occurred despite the Italian colonies having overall larger populations, more brood, and a marginally earlier start to foraging activity in the day.

Additional evidence highlighting genetic contributions to colony performance comes from Alburaki and Garnery, who observed that mitochondrial haplotypes associated with ligustica (C1) and carnica (C2) maternal provenance correlated with differences in thermoregulation and colony weight gain [52]. These results suggest that genetic lineage can meaningfully shape both physiological and behavioural traits relevant to foraging efficiency.

Collectively, our findings support the interpretation that genetic background influences foraging productivity, possibly through traits related to thermoregulation, behavioural responsiveness, disease resistance, and colony cohesion. However, because our analysis of genetic effects is limited to a single year and specific environmental context, we caution that broader generalisations require additional multi-year and multi-environment trials.

### 4.4. Colony History and Management Effects

A plausible explanation for the comparatively weaker performance of unselected stock bees in the 2023 almond study could lie in the fact that the stronger colonies in this group were obtained by combining smaller units. Although combining colonies is a useful and standard beekeeping practice, the short-term consequences of such interventions are not well documented. A temporary reduction in colony-level cohesion may occur, which could negatively affect productivity in the short term.

Historical studies by Farrar lend support to this hypothesis: he found that package bees were significantly less efficient as pollination units compared to both overwintered and newly raised nucleus colonies [25,53]. Farrar concluded that if package bees are to be used for pollination, they should be established well in advance of deployment.

Given that we lack detailed behavioural data from the immediate post-combination period in our study, we cannot quantify the magnitude of this effect. However, the consistency of Farrar’s findings with the underperformance observed in our unselected stock colonies suggests that the colony formation method may be an important covariate in pollination efficiency studies based on colony performance and merits systematic investigation in future work.

### 4.5. Colony Performance During Forage Dearth

In both phenological and short-term weather contexts, periods of unfavourable foraging conditions typically resulted in net colony weight loss, and our results show that stronger colonies lost relatively less weight than weaker colonies. This raises questions as to whether larger colonies demonstrate greater metabolic efficiency under resource scarcity, and which factors contribute to this.

Robinia typically offers a heavy nectar flow and marks the peak of colony development in the Apennine foothills of Central Italy. However, during our study, weather conditions at the onset of bloom were unstable, disrupting both bee activity and nectar production. Due to the large size of colonies at that time, breaks in forage availability resulted in weight losses driven by sharp increases in the consumption of in-hive stored resources. Low nighttime temperatures at this time of year increase a colony’s metabolic demand as it maintains temperature homeostasis. Our Robinia 2025 dataset yielded a significant negative relationship between colony strength and weight loss per FOB (R^2^ = 0.60, *p* = 0.02), indicating that stronger colonies were better able to limit proportional weight losses during this period.

During the post-bloom period of the 2024 almond study, the pronounced floral dearth that typically follows monoculture bloom led to the highest colony weight losses recorded in all our studies. Furthermore, low ambient nighttime temperatures characteristic of this period likely increased the metabolic demand and consequent consumption of stored food. Our results for this period once more show that stronger colonies lost less weight per FOB, with a significant negative relationship between colony strength and weight loss per FOB (R^2^ = 0.56, *p* = 0.031). These results reinforce the case that colony strength enhances resilience when environmental stressors compound.

Our 2025 dataset provided an opportunity to analyse colony performance during summer dearth between Robinia and sweet chestnut bloom, two major nectar flows of the region. As observed in Robinia and almonds 2024, stronger colonies lost less weight per FOB than weaker ones; however, this trend was not statistically significant (R^2^ = 0.38, *p* = 0.102). Unlike earlier periods, ambient temperatures at this time of year were mild and stable, reducing metabolic weight losses attributable to thermoregulation.

These findings indicate that the benefits of colony strength during dearth are most pronounced when forage scarcity coincides with elevated thermoregulation costs at cooler temperatures. This interpretation aligns with physiological research showing that thermoregulation is more energy efficient in larger colonies, as heat production and retention scale with the number of bees [54,55,56]. While Free and Racey found that smaller colonies consumed less honey per bee [57], Harbo observed that larger colonies lost less weight per bee during winter as well as during summer dearth [28].

Behavioural mechanisms may also contribute to the increased efficiency of larger colonies during summer dearth, namely the more efficient exploitation of food resources over greater distances when forage is scarce [40,42]. Although they examined different bee species, both Grüter and Hayes [58] and Kendall et al. [59] present evidence that foraging distances increase with colony size during periods of limited forage. Donaldson-Matasci et al. also showed that larger colonies benefit more from communication than smaller colonies and, as a result, honey bees in larger colonies were more effective foragers [42].

Taken together, these physiological (thermoregulatory) and behavioural (foraging) mechanisms may confer a disproportionate advantage to strong colonies in food-limited environments, thus mitigating weight losses relative to colony strength. While there is evidence that colony size does not significantly affect thermoregulatory *effectiveness*, as honey bee colonies maintain remarkably stable brood temperatures regardless of population size [60], our results suggest that the *efficiency* of thermoregulation may nonetheless vary with colony size. We propose that stronger colonies may be better able to buffer energetic costs on a per-FOB basis.

The sole exception to the observed weight losses occurred in the 2023 almond study. Exceptionally poor weather during early almond bloom disrupted not only colony activity but also compromised weight measurements, rendering the analysis of losses unfeasible. Nonetheless, as meteorological conditions improved, we noted that colonies continued to gain weight, both toward the end of almond bloom and immediately thereafter, with the largest weight gains observed following bloom. Upon further investigation of alternative forage in the surrounding area, we discovered high bee activity on extensive annual flowering cover crops (phacelia and mustard) within one kilometre of the study site. The following year (2024), these fields were re-planted with crops that did not offer floral resources to bees. These findings add to the growing body of evidence that cover crops benefit managed pollinators in highly simplified agricultural landscapes [61,62,63].

### 4.6. Queen Age and Colony Performance

In all of our studies, most colonies were headed by one-year-old queens, with the exception of two colonies in the Robinia and sweet chestnut (2025) studies, which had same season queens. During the Robinia study, those two colonies were part of the Weak group, yet their relative gains (per FOB) were higher than any other colony in the study, including the Strong group. Likewise, during dearth, they lost less weight per FOB than any other colony, making them the most efficient colonies in the Robinia study. By the time sweet chestnut bloomed, those same colonies had increased in size considerably and moved into the Strong group. Interestingly, in the sweet chestnut study they gained more weight in both absolute (per colony) and relative (per FOB) terms; they also lost less relative weight than any other colony. Their performance suggests that colonies headed by new queens may undergo a period of enhanced productivity and efficiency.

Previous studies have shown that queen age can positively affect colony productivity [64,65,66,67]. In contrast, Szabo and Lefkovitch reported no differences in productivity, but their work compared colonies with 2- and 3-year-old queens [31]. As none of these studies investigate the effect of same season queens, and our sample size is low, this topic justifies further investigation.

Taken together, our observations suggest that queen age may positively influence colony efficiency, particularly during periods of rapid colony expansion. However, given the limited sample, further research is needed to determine whether the superior performance of colonies headed by new queens reflects a consistent biological phenomenon, a temporary early-development effect, or a context-dependent interaction with environmental conditions.

## 5. Conclusions

Based on our results, we propose that strong colonies confer a non-linear advantage in efficiency across a wide range of environmental contexts. Colony strength improves both the gains and resilience to losses until the advantage plateaus at very high strengths. This efficiency advantage is particularly evident under unfavourable environmental conditions, where stronger colonies deliver more consistent pollination, thus helping to mitigate the risk of pollination deficit for growers. Beekeepers likewise benefit, as strong colonies capitalise on resources more efficiently and effectively and therefore leave the almond orchards much stronger, kick-starting the beekeeping season and potentially improving outcomes for the rest of the year.

Importantly, using high stocking densities to compensate for weak colonies can negatively affect honey bee health. High colony densities have been linked to increased disease, including European foul brood (EFB), as reported by Eeraerts et al. in blueberry systems [68]. Such disease pressures undermine beekeepers’ margins and can reduce long-term willingness to supply pollination services for high-value crops. Further support for deploying stronger colonies can be found in their increased foraging ranges [41], which allow greater deployment flexibility, both in terms of location and distribution of pollinating units. Furthermore, with fewer hives to load, transport, and deploy, the logistical burden is reduced, leading to savings in labour and transportation while minimising disruption to the operation of farm machinery within the orchard. Finally, the ensuing reduction in transportation lessens the environmental burden in terms of both fuel consumption and emissions, making pollination service provision a more sustainable business.

## Figures and Tables

**Table 1:** Comparison of weight gain and efficiency between Weak and Strong honey bee colony groups across three crops and three years.

Study	Colony Group (Mean FOB)	Mean Weight Gain ± SE (kg)	Mann–Whitney U	z	*p* (Two Tailed)	Efficiency (Strong/Weak)
Almond (*Prunus dulcis*) 2023	Weak (2.28)	1.20 ± 0.93	38	−1.72	0.085 ns	2.63
	Strong (6.26)	5.09 ± 1.77				
Almond (*Prunus dulcis*) 2024	Weak (6.57)	4.05 ± 1.04	3	−2.57	0.010 *	2.20
	Strong (11.17)	12.08 ± 1.64				
Robinia (*Robinia pseudoacacia*) 2025	Weak (4.57)	5.30 ± 1.08	0	−2.61	0.009 **	1.19/1.92
	Strong (10.35)	14.28 ± 0.62				
Sweet chestnut (*Castanea sativa*) 2025	Weak (4.73)	10.36 ± 2.09	1	−2.4	0.016 *	1.3
	Strong (7.25)	19.61 ± 2.04				

Efficiency is calculated as the ratio of mean weight gain per unit colony strength (kg FOB^−1^) in Strong colonies relative to Weak colonies within each study period. Mann–Whitney U tests compare absolute colony weight gains between Weak and Strong groups within each study period. The U statistic, corresponding standardised z value, and associated two-tailed *p* value are reported. Colony strength groups were defined by the median frames of bees (FOBs) within each study. Mean FOB values for each group are shown in parentheses. *p* values are shown as exact calculated probabilities. Significance levels: *p* < 0.05 (*), *p* < 0.01 (**), ns = not significant.

## Data Availability

The raw data supporting the conclusions of this article are provided in the Supplementary Materials (Supplementary File S1).

## Supplementary Materials

The following supporting information can be downloaded at https://www.mdpi.com/article/10.3390/insects17020163/s1. The raw data used to generate the results of this study are provided as Supplementary File S1.

## References

[1] Sáez A., Aguilar R., Ashworth L., Gleiser G., Morales C.L., Traveset A., Aizen M.A. (2022). Managed honeybees decrease pollination limitation in self-compatible but not in self-incompatible crops. Proc. Biol. Sci..

[2] Aizen M.A., Garibaldi L.A., Cunningham S.A., Klein A.M. (2009). How much does agriculture depend on pollinators? Lessons from long-term trends in crop production. Ann. Bot..

[3] Aizen M.A., Aguiar S., Biesmeijer J.C., Garibaldi L.A., Inouye D.W., Jung C., Martins D.J., Medel R., Morales C.L., Ngo H. (2019). Global agricultural productivity is threatened by increasing pollinator dependence without a parallel increase in crop diversification. Glob. Change Biol..

[4] Osterman J., Landaverde-González P., Garratt M.P.D., Gee M., Mandelik Y., Langowska A., Miñarro M., Cole L.J., Eeraerts M., Bevk D. (2021). On-farm experiences shape farmer knowledge, perceptions of pollinators, and management practices. Glob. Ecol. Conserv..

[5] Dicks L.V., Breeze T.D., Ngo H.T., Senapathi D., An J., Aizen M.A., Basu P., Buchori D., Galetto L., Garibaldi L.A. (2021). A global-scale expert assessment of drivers and risks associated with pollinator decline. Nat. Ecol. Evol..

[6] Koh I., Lonsdorf E.V., Williams N.M., Brittain C., Isaacs R., Gibbs J., Ricketts T.H. (2016). Modeling the status, trends and impacts of wild bee abundance in the United States. Proc. Natl. Acad. Sci. USA.

[7] Senapathi D., Goddard M.A., Kunin W.E., Baldock K.C.R. (2017). Landscape impacts on pollinator communities in temperate systems: Evidence and knowledge gaps. Funct. Ecol..

[8] Kennedy C.M., Lonsdorf E., Neel M.C., Williams N.M., Ricketts T.H., Winfree R., Bommarco R., Brittain C., Burley A.L., Cariveau D. (2013). A global quantitative synthesis of local and landscape effects on wild bee pollinators in agroecosystems. Ecol. Lett..

[9] Cook D., Tarlinton B., McGree J.M., Blackler A., Hauxwell C. (2022). Temperature sensing and honey bee colony strength. J. Econ. Entomol..

[10] Cunningham S.A., Fournier A., Neave M.J., Le Feuvre D. (2016). Improving spatial arrangement of honeybee colonies to avoid pollination shortfall and depressed fruit set. J. Appl. Ecol..

[11] Potts S.G., Imperatriz-Fonseca V.L., Ngo H.T., Biesmeijer J.C., Breeze T.D., Dicks L.V., Garibaldi L.A., Hill R., Settele J., Vanbergen A.J., IPBES (2016). Summary for Policymakers of the Assessment Report of the Intergovernmental Science-Policy Platform on Biodiversity and Ecosystem Services on Pollinators, Pollination and Food Production.

[12] Velthuis H.H.W., van Doorn A. (2006). A century of advances in bumblebee domestication and the economic and environmental aspects of its commercialization for pollination. Apidologie.

[13] Potts S.G., Imperatriz-Fonseca V., Ngo H.T., Aizen M.A., Biesmeijer J.C., Breeze T.D., Dicks L.V., Garibaldi L.A., Hill R., Settele J. (2016). Safeguarding pollinators and their values to human well-being. Nature.

[14] Garibaldi L.A., Steffan-Dewenter I., Winfree R., Aizen M.A., Bommarco R., Cunningham S.A., Kremen C., Carvalheiro L.G., Harder L.D., Afik O. (2013). Wild pollinators enhance fruit set of crops regardless of honey bee abundance. Science.

[15] Yeh D., Brown C., Goodrich B. (2025). Buzzing toward sustainability: Protecting pollinators to strengthen specialty crop production. Choices Mag..

[16] Rucker R.R., Thurman W.N., Burgett M. (2012). Honey bee pollination and the internalization of reciprocal benefits. Am. J. Agric. Econ..

[17] Fenton M., Goodrich B. (2023). Economic considerations of growing bee-friendly cover crops in almond orchards: Grower and beekeeper perspectives. ARE Update.

[18] Goodrich B., Altschuler A. (2025). Where have all the honey bees gone? To California almond orchards. Farmdoc Dly..

[19] Almond Board of Australia (2022). Honey Bee Best Management Practices for Almond Pollination.

[20] Simone-Finstrom M., Strand M.K., Tarpy D.K., Rueppell O. (2022). Impact of Honey Bee Migratory Management on Pathogen Loads and Immune Gene Expression is Affected by Complex Interactions with Environment, Worker Life History, and Season. J. Insect Sci..

[21] Wu S., Liu J., Lei X., Zhao S., Lu J., Jiang Y., Xie B., Wang M. (2022). Research Progress on Efficient Pollination Technology of Crops. Agronomy.

[22] Haviland D.R., Yaghmour M., Fichtner E.J., Mireles R., Culumber M., Murdock J., Long P. (2024). Sample Costs to Produce Almonds, San Joaquin Valley, South (Double-line Drip Irrigation), 2024.

[23] Veldtman R. (2018). Are managed pollinators ultimately linked to the pollination ecosystem service paradigm?. S. Afr. J. Sci..

[24] Evans S.K., Evans H., Meikle W.G., Clouston G. (2024). Hive orientation and colony strength affect honey bee colony activity during almond pollination. Insects.

[25] Farrar C.L. (1931). The evaluation of bees for pollination. J. Econ. Entomol..

[26] Woodrow A.W. (1932). The comparative value of different colonies of bees in pollination. J. Econ. Entomol..

[27] Harbo J.R. (1983). Effect of population size on worker survival and honey loss in broodless colonies of honey bees, *Apis mellifera* L. (Hymenoptera: Apidae). Environ. Entomol..

[28] Harbo J.R. (1986). Effect of population size on brood production, worker survival and honey gain in colonies of honeybees. J. Apic. Res..

[29] Woyke J. (1984). Correlations and interactions between population, length of worker life and honey production by honeybees in a temperate region. J. Apic. Res..

[30] Sheesley R., Poduska B.E. (1970). Strong honeybee colonies prove value in almond pollination. Calif. Agric..

[31] Szabo T.I., Lefkovitch L.P. (1989). Effect of brood production and population size on honey production of honeybee colonies in Alberta, Canada. Apidologie.

[32] Zacepins A., Ozols N., Kviesis A., Gailis J., Komasilovs V., Komasilova O., Zagorska V. (2022). Evaluation of honey bee colony weight gain during the intensive foraging period. Agron. Res..

[33] Evans H., Stanisavljević L. (2022). Development of in-field acoustic sensors for monitoring pollinator visitation rates. Book of Abstracts from 9th European Congress of Apidology (EurBee9), Belgrade, Serbia, 20–22 September 2022.

[34] Dainat B., Dietemann V., Imdorf A., Charrière J.D. (2020). A scientific note on the “Liebefeld Method” to estimate honey bee colony strength: Its history, use, and translation. Apidologie.

[35] Woodrow A.W. (1934). The effect of colony size on the flight rates of honeybees during the period of fruit bloom. J. Econ. Entomol..

[36] Danka R.G., Rinderer T.E., Hellmich R.L., Collins A.M. (1986). Foraging population sizes of Africanized and European honey bee (*Apis mellifera* L.) colonies. Apidologie.

[37] Farrar C.L. (1937). The influence of colony populations on honey production. J. Agric. Res..

[38] Woodrow T. (1933). The Comparative Value of Different Colonies of Bees for Fruit Pollination.

[39] Free J.B. (1967). Factors determining the collection of pollen by honeybee foragers. Anim. Behav..

[40] Beekman M., Sumpter D.J.T., Seraphides N., Ratnieks F.L.W. (2004). Comparing foraging behaviour of small and large honey-bee colonies by decoding waggle dances made by foragers. Funct. Ecol..

[41] Eckert C.D., Winston M.L., Ydenberg R.C. (1994). The relationship between population size, amount of brood, and individual foraging behaviour in the honey bee, *Apis mellifera* L.. Oecologia.

[42] Donaldson-Matasci M.C., DeGrandi-Hoffman G., Dornhaus A. (2013). Bigger is better: Honeybee colonies as distributed information-gathering systems. Anim. Behav..

[43] Gary N.E., Witherell P.C., Marston J.M. (1978). Distribution and foraging activities of honeybees during almond pollination. J. Apic. Res..

[44] Danka R.G., Sylvester H.A., Boykin D. (2006). Environmental influences on flight activity of USDA–ARS Russian and Italian stocks of honey bees during almond pollination. J. Econ. Entomol..

[45] Michener C.D. (1964). Reproductive efficiency in relation to colony size in hymenopterous societies. Insectes Soc..

[46] Jeanne R.L., Loope K.J., Bouwma A.M., Nordheim E.V., Smith M.L. (2022). Five decades of misunderstanding in the social Hymenoptera: A review and meta-analysis of Michener’s paradox. Biol. Rev..

[47] Rivera M.D., Donaldson-Matasci M., Dornhaus A. (2015). Quitting time: When do honeybee foragers decide to stop foraging on natural resources?. Front. Ecol. Evol..

[48] Higo H.A., Colley S.J., Winston M.L., Slessor K.N. (1992). Effects of honey bee (*Apis mellifera* L.) queen mandibular gland pheromone on foraging and brood rearing. Can. Entomol..

[49] Pankiw T., Page R.E., Fondrk M.K. (1998). Brood pheromone stimulates pollen foraging in honey bees (*Apis mellifera*). Behav. Ecol. Sociobiol..

[50] Free J.B., Preece D.A. (1969). The effect of the size of a honeybee colony on its foraging activity. Insectes Soc..

[51] Meikle W.G., Weiss M., Ricigliano V.A. (2025). Continuous hive monitoring reveals colony growth and activity differences among mite-resistant and Italian honey bee stocks. Apidologie.

[52] Alburaki M., Garnery L. (2024). Effects of landscape variation on thermoregulation and performance in *Apis mellifera* honey bee colonies: Insights from mtDNA haplotypes. J. Apic. Res..

[53] Farrar C.L. (1936). Influence of pollen reserves on the surviving population of overwintered colonies. Am. Bee J..

[54] Seeley T.D., Heinrich B., Heinrich B. (1981). Regulation of temperature in the nests of social insects. Insect Thermoregulation.

[55] Southwick E.E. (1983). The honey bee cluster as a homeothermic superorganism. Comp. Biochem. Physiol. A Physiol..

[56] Southwick E.E. (1985). Allometric relations, metabolism and heart conductance in clusters of honey bees at cool temperatures. J. Comp. Physiol. B.

[57] Free J.B., Racey P.A. (1968). The effect of the size of honeybee colonies on food consumption, brood rearing and the longevity of the bees during winter. Entomol. Exp. Appl..

[58] Grüter C., Hayes L. (2022). Sociality is a key driver of foraging ranges in bees. Curr. Biol..

[59] Kendall L.K., Mola J.M., Portman Z.M., Cariveau D.P., Smith H.G., Bartomeus I. (2022). The potential and realized foraging movements of bees are differentially determined by body size and sociality. Ecology.

[60] Godeau U., Pioz M., Martin O., Rüger C., Crauser D., Le Conte Y., Henry M., Alaux C. (2023). Brood thermoregulation effectiveness is positively linked to the amount of brood but not to the number of bees in honeybee colonies. Peer Community J..

[61] Mallinger R.E., Franco J.G., Prischmann-Voldseth D.A., Prasifka J.R. (2019). Annual cover crops for managed and wild bees: Optimal plant mixtures depend on pollinator enhancement goals. Agric. Ecosyst. Environ..

[62] Mayack C., Carlson M., Niño B.D., Niño E.L., Seshadri A. (2025). Impacts of almond pollination service and inter-row cover cropping on honey bee colony strength and performance. Sci. Total Environ..

[63] Wauters V.M., Jarvis-Shean K., Williams N., Hodson A., Hanson B.D., Haring S., Gaudin A.C.M. (2023). Developing cover crop systems for California almonds: Current knowledge and uncertainties. J. Soil Water Conserv..

[64] Farrar C.L. (1944). Productive Management of Honeybee Colonies in the Northern States.

[65] Simeunovic P., Stevanovic J., Cirkovic D., Radojicic S., Lakic N., Stanisic L., Stanimirovic Z. (2014). *Nosema ceranae* and queen age influence the reproduction and productivity of the honey bee colony. J. Apic. Res..

[66] Hauser R., Lensky Y. (1994). The effect of the age of the honey bee (*Apis mellifera* L.) queen on worker population, swarming and honey yields in a subtropical climate. Apidologie.

[67] Akyol E., Yeninar H., Kaftanoglu O. (2008). Live weight of queen honey bees (*Apis mellifera* L.) predicts reproductive characteristics. J. Kans. Entomol. Soc..

[68] Eeraerts M., Rogers E., Gillespie B., Best L., Smith O.M., DeVetter L.W. (2023). Landscape-level honey bee hive density, instead of field-level hive density, enhances honey bee visitation in blueberry. Landsc. Ecol..

